# Facial feature removal in magnetic resonance imaging scans of adults with Down syndrome: A de‐facing methodological study

**DOI:** 10.1002/alz.71614

**Published:** 2026-06-26

**Authors:** Jason K. Russell, Michael S. Rafii, Walter K. Kremers, Scott A. Przybelski, Prashanthi Vemuri, Ronald C. Petersen, Jonathan Graff‐Radford, Clifford R. Jack, Christopher G. Schwarz

**Affiliations:** ^1^ Alzheimer's Therapeutic Research Institute, Keck School of Medicine University of Southern California San Diego California USA; ^2^ Department of Quantitative Health Sciences, Division of Biostatistics Mayo Clinic Rochester Minnesota USA; ^3^ Department of Radiology, Mayo Clinic Rochester Minnesota USA; ^4^ Department of Neurology, Mayo Clinic Rochester Minnesota USA

**Keywords:** de‐facing, de‐identification, Down syndrome, magnetic resonance imaging (MRI), mri_reface, neuroimaging

## Abstract

**INTRODUCTION:**

Face identification algorithms have increased the risk of study participants being identified through different neuroimaging modalities. De‐facing algorithms can de‐identify neuroimages; however, none are validated in individuals with Down syndrome (DS) – a population at high risk of Alzheimer's disease and the focus of numerous Alzheimer's disease cohort studies.

**METHODS:**

Overall, 37 adults with DS were parcellated using FreeSurfer before and after de‐facing with *mri_reface*. A group of neurotypically developed individuals balanced on age, sex, and magnetic resonance imaging scanner manufacturer served as controls.

**RESULTS:**

De‐facing produced no effect on cortical thickness or volumetrics in adults with DS compared to controls or regional discrimination based on an area under the receiver operating characteristic curve analysis after correcting for multiple comparisons.

**DISCUSSION:**

FreeSurfer‐derived volumetric and cortical thickness measures changed minimally following de‐facing with *mri_reface* in adults with DS and are unlikely to influence study outcomes. De‐facing should be considered prior to data sharing in studies of adults with DS.

## Highlights

- This is the first assessment of any de‐facing methodology in DS.
- De‐facing can be used to protect privacy in DS studies.
- De‐facing with *mri_reface* has negligible effects on brain measurements in DS.
- De‐facing with *mri_reface* should be considered for DS studies with data sharing.

## 1 BACKGROUND

Increasingly sophisticated face recognition technologies employing convolutional neural networks and deep learning have been developed over the last 10 years, increasing the risk of research participants being re‐identified using facial imagery from multiple neuroimaging modalities.^1^, ^2^ Previous reports suggested that automatic matching of photographs to magnetic resonance imaging (MRI) could be up to 98% accurate, with positron emission tomography (PET) up to 42% accurate and computed tomography (CT) up to 78% to 83% accurate.^1^, ^2^, ^3^ These privacy concerns have fueled the development of improved methods to de‐identify neuroimages through either face removal or face replacement.^4^ Recent studies in sporadic Alzheimer's disease (AD) and aging populations have featured *mri_reface*, a software that replaces the original face image with a population average face image and produces minimal impact on neuroimaging measures when compared to alternate methods.^4^, ^5^, ^6^, ^7^, ^8^

Individuals with DS (i.e., trisomy 21) have an extra copy of the *APP* gene found on chromosome 21, producing a 95% lifetime risk of developing AD.^9^ This has resulted in numerous studies focusing on the development of AD‐related pathology in this unique genetically determined population.^10^, ^11^, ^12^, ^13^, ^14^ Large multi‐modal imaging studies performed in adults with DS include those where neuroimaging data are shared with the broader scientific community.^15^ Given the increasing privacy concerns associated with sharing neuroimaging data, consideration should be given to appropriate de‐identification of study images prior to data sharing. Numerous de‐facing algorithms are available to the scientific community;^16^, ^17^, ^18^, ^19^ however, individuals with DS display different craniofacial morphology compared to neurotypically developed (TD) individuals,^20^ which may adversely influence the performance of existing de‐facing algorithms.

In this study, we sought to validate the *mri_reface* algorithm, developed with a population average face for sporadic AD and aging, in adults with DS, by assessing FreeSurfer‐derived volumetric and thickness measures before and after de‐facing in participants from the Trial Ready Cohort—Down Syndrome (TRC‐DS).^21^ The Mayo Clinic Study of Aging (MCSA) is a population‐based cohort study of individuals from 30 years and older,^22^ which is utilized as a matched comparator group and to assess whether age introduces bias to *mri_reface* performance.

**RESEARCH IN CONTEXT**

1. **Systematic review**: PubMed was searched from database inception to September 9, 2025, for de‐facing, de‐identification in neuroimaging, and DS. Previous studies investigated the utility of numerous de‐facing algorithms in aging and AD; however, none of these methods have been validated in individuals with DS, who display different craniofacial morphology that may influence the performance of these algorithms.
2. **Interpretation**: This study of 37 adults with DS provides validation of the *mri_reface* algorithm in adults with DS indicating its use should be considered in studies of individuals with DS prior to data sharing.
3. **Future directions**: This analysis includes only adults with DS. Further studies will be required to assess the validity of these methods in younger individuals with and without DS.

## 2 METHODS

### 2.1 Study design and participants

Individuals with DS were recruited as part of a multicenter longitudinal observation cohort study (TRC‐DS) at sites in the United States and Europe from July 2021 to December 2024. Participants were required to be 25 to 55 years old inclusive and not have a diagnosis of dementia. Participants were excluded if they had an unstable medical condition, contraindications to MRI, any conditions that would preclude neuropsychiatric testing, abnormalities in B12 or thyroid function tests, previously receiving a radiation dose exceeding 30 mSv, or concurrent enrollment in a clinical trial or longitudinal study with overlapping measures or prohibited procedures. For this analysis, to facilitate group‐level age and scanner balancing with MCSA, TRC‐DS participants were excluded if they were under 30 years old (*n* = 16) or if a Philips MRI scanner was used for data collection, due to the fact that Philips scanners are not utilized in MCSA (*n* = 26, including five previously excluded for age <30), resulting in 41 potential participants for assessment. However, four participants were excluded because either their FreeSurfer parcellations (*n* = 2) or their de‐facing (*n* = 2) did not pass visual quality control (QC), which left 37 TRC‐DS individuals for final analyses. All FreeSurfer parcellation failures were in participants with significant movement in the unedited scan, which would typically be excluded due to poor image quality.

Cognitively unimpaired participants were selected from the MCSA, a prospective, longitudinal population‐based cohort study of individuals ages 30+ from Olmsted County, Minnesota, USA,^22^ to create a matched group for the TRC‐DS cohort based on age, sex, and MRI scanner manufacturer (matched roughly 2:1, TD individuals to individuals with DS, MCSA‐matched cohort). Because the average template face used in *mri_reface* was developed using images from individuals who were much older than those in typical DS cohorts, we also selected a second cohort of 470 individuals from the MCSA with a uniform distribution of ages 31 to 89, to assess whether *mri_reface* would have different effects in younger individuals than older ones (MCSA age range cohort).

### 2.2 Procedures

Participants received three‐dimensional T1‐weighted MRI scans performed as part of the standard procedures for TRC‐DS or MCSA. MRI scans were performed on Siemens or GE MRI scanners (for TRC‐DS, this depended on the acquisition site; for MCSA, this depended on the acquisition date before/after a protocol change) and balanced across groups based on manufacturer (Table 1). Where participants received multiple scans, the scan at the earliest visit with acceptable quality control assessments was selected for analysis. MRI scans were visually assessed for movement artifacts. For TRC‐DS, MRI scans were T1‐weighted accelerated magnetization prepared rapid gradient echo (Siemens) or inversion recovery – fast spoiled gradient recalled echo (GE) sequences, with harmonized resolution across sites (∼1‐mm in‐plane and 1.2‐mm slice thickness). For Siemens scanners, acquisition parameters were repetition time (TR) = 2300 ms, echo time (TE) = ∼2.9 ms, inversion time (TI) = ∼900 ms, and flip angle = 9°. For GE scanners, TR = ∼7.6 to 7.7 ms, TE = ∼3.1, TI = 400 ms, and flip angle 11°.

**TABLE 1:** Participant demographics.

	Adults with Down syndrome	Age‐matched neurotypically developed controls	*p* value	Older‐aged neurotypically developed controls
N	37	78	N/A	470
Age (years)				
Mean (range)	39.9 (30–55)	40.8 (31–55)	0.525	60.1 (30–89)
Sex (%)			0.982	
Female	16 (43.2%)	32 (41.0%)		235 (50%)
Male	21 (56.8%)	46 (59.0%)		235 (50%)
ID (%)			NA	
Mild	20 (54.1%)	NA		NA
Moderate	14 (37.8%)	NA		NA
Severe	3 (8.1%)	NA		NA
mCRT TRS (SD)	34.2 (4.2)	NA	NA	NA
DSMSE (SD)	80.8 (12.7)	NA	NA	NA
MRI manufacturer			0.938	
Siemens	20 (54.1%)	40 (51.3%)		230 (48.9%)
GE	17 (45.9%)	38 (48.7%)		240 (51.1%)

*Note*: Age compared between adults with DS and age‐matched neurotypically developed individuals using a *t*‐test. Sex and MRI manufacturer compared between adults with DS and age‐matched neurotypically developed individuals using chi‐squared test.

Abbreviations: DSMSE, Down syndrome mental state exam total score; ID, premorbid intellectual disability; mCRT TRS, modified cued recall score total recall score; MRI, magnetic resonance imaging; SD, standard deviation.

### 2.3 MRI quantification

MRI scans were processed prior to and following de‐facing procedures using *recon‐all* in FreeSurfer 7.4.1.^23^ Standard volumes and cortical thicknesses from the default Desikan‐Killiany parcellation were compared before and after de‐facing to assess the effect of de‐facing on adults with DS.^24^ For TRC‐DS participants, FreeSurfer outputs were visually assessed to confirm accurate parcellation. If a participant's scan had high levels of motion and poor parcellation, a follow‐up MRI scan from that participant was selected as a replacement, if available and of sufficient quality. This resulted in two participants (previously mentioned) being excluded from the final analysis. For MCSA, only images that passed QC for motion and artifacts were processed; given the negligible failure rate in high‐quality scans of cognitively unimpaired, relatively young participants, visual QC was not performed.

### 2.4 De‐facing

**FIGURE 1:**
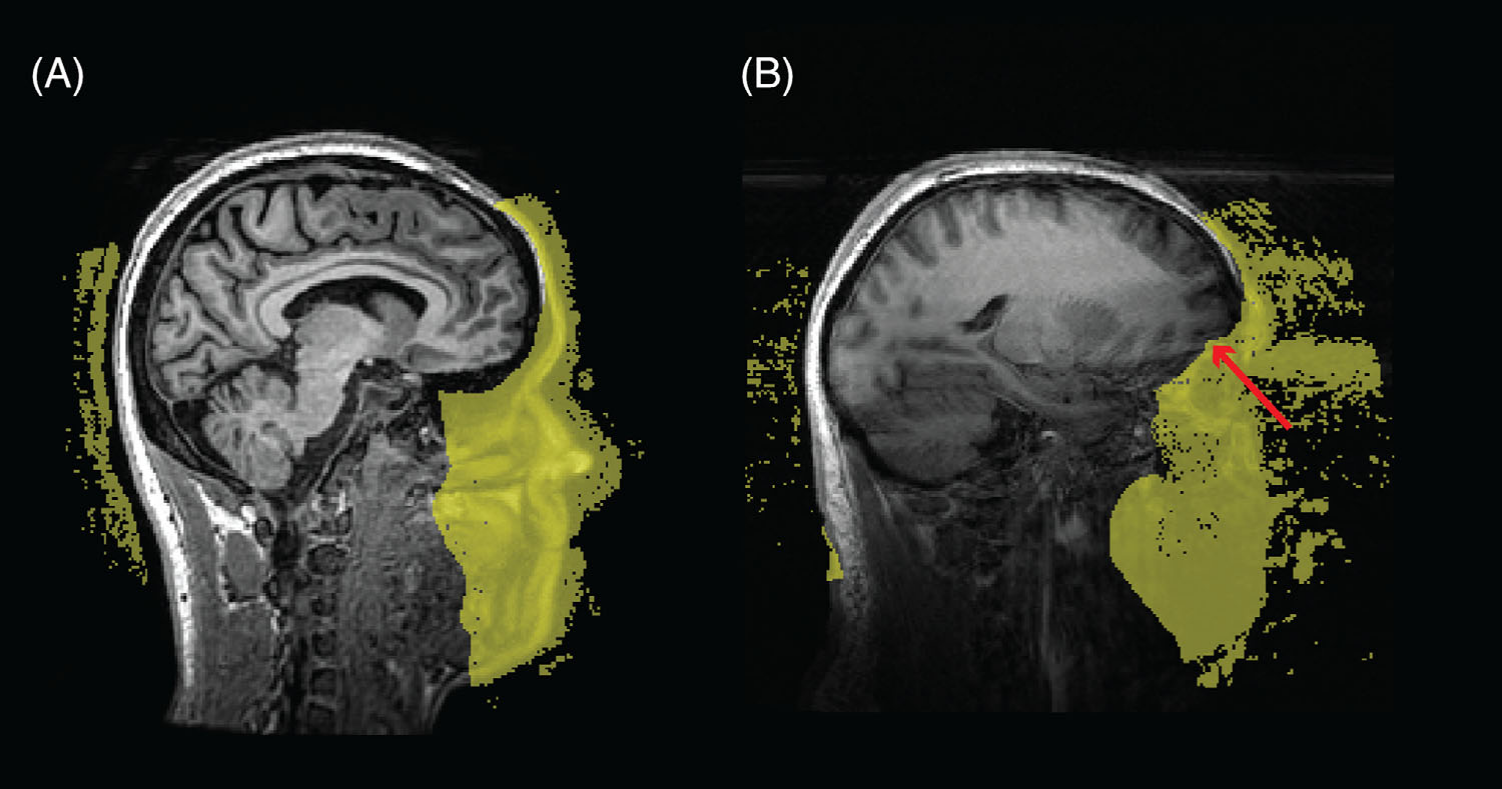
Representative examples of de‐facing with *mri_reface*. Shown are the MRIs from two individuals with DS. The yellow overlay indicates voxels that were altered by the de‐facing processes. (A) Image with appropriate face replacement with no brain alteration. (B) Image where a small amount of brain tissue was altered, highlighted by red arrow. This occurred in only two cases, and they were excluded from the analyses.

Participants’ MRI scans were de‐faced with *mri_reface* 0.3.5 (https://www.nitrc.org/projects/mri_reface) using default parameters following previously described methods.^16^ The de‐facing process was visually assessed in the TRC‐DS and MCSA‐matched group to confirm adequate face replacement and no visual alteration of the brain. Face renders generated by *mri_reface* were compared before and after de‐facing to confirm the face had been adequately replaced, and the voxels that had been changed by the de‐facing process were evaluated to assess whether any brain voxels had been edited. Two individuals with DS failed this visual quality control, with voxels from the brain altered. These were corrected by slightly increasing the “TIVToleranceOffset” option of *mri_reface*, but for the purpose of standardizing all parameters for this comparison, they were instead excluded (previously mentioned) from the analyses (Figures 1 and S1).

### 2.5 Statistical analysis

Statistical analyses were performed using R version 4.3.2. To assess group differences between the DS and MCSA‐matched groups in age, sex, and MRI scanner manufacturer (Table 1), we used *t*‐tests for age and chi‐squared test for proportions of sex and MRI scanner manufacturer. To assess differences in de‐facing performance across the available adult age range (MCSA age range cohort), we uniformly sampled MCSA participants and ran FreeSurfer on each image both before and after *mri_reface*. For each regional measurement of tissue volume or cortical thickness, we subtracted the measurement on the de‐faced image from the measurement on the unmodified image, producing a simple subtractive difference value. We then used a linear model F‐test for each regional measurement to assess the significance of age in predicting these measurement differences caused by de‐facing when controlling for the effects of sex and scanner manufacturer. To assess whether the measurement differences due to de‐facing were different between TD adults (MCSA‐matched cohort) and adults with DS (TRC‐DS), we performed the same steps of running FreeSurfer on each image before and after *mri_reface* and calculating subtractive differences for each measure. We then performed linear model F‐tests to assess the significance of the group variable (DS vs TD), while controlling for the effects of age, sex, and MRI scanner manufacturer. We considered this our primary analysis for the current study. We also examined how de‐facing affected the biomarker performance of each regional measurement by performing a receiver operating characteristic (ROC) curve analysis (pROC package in R) and calculating each measurement's area under the ROC curve (AUROC) for classifying DS versus TD. For all volumetric measurements in the AUROC analysis (only), we first divided each value by FreeSurfer's estimated total intracranial volume (TIV) to adjust for differences in head size, as is typical for volumetric biomarker measurements. We used DeLong's method (pROC::roc.test(paired = true)) to test for significant differences between each region's ROC curve with unmodified images versus each region's ROC curve with de‐faced images. Standard ROC curve analyses are ubiquitous in the biomarker literature and interpretable to scientists in this field, but they do not allow for adjusting for covariates, so our ROC curve analyses did not adjust for differences in age, sex, or scanner manufacturer; however, our groups were already balanced with minimal differences between them, and the ROC curve analyses are considered secondary and partly redundant with our primary analyses that did adjust for these covariates. We considered *p* values < 0.05 to be statistically significant. To perform correction for multiple comparisons across the roughly 150 regional FreeSurfer measurements, for each of our three analyses (age, DS direct differences, and DS AUCs) we used permutation methods to calculate family‐wise *p* values adjusted for multiple comparisons based on 10,000 permutations. Although these regional measurements also included hemispheric (thickness) and whole‐brain (volume) composite measures, these composites are part of the measurements produced by FreeSurfer alongside the individual region measurements, and so we included them with the rest in our corrections for multiple comparisons, even though they are not independent.

## 3 RESULTS

### 3.1 Demographics

A total of 37 adults with DS, average age 39.9 years old (range 30 to 55; 21 males, 16 females), and 78 TD individuals in the age‐, sex‐, and scanner‐balanced group, average age 40.8 (range 31 to 55; 46 males, 32 females) were included in the final analysis. Overall, 470 TD individuals, average age 60.1 (range 30 to 89; 235 males, 235 females) were analyzed to assess the effects of age on de‐facing. There were no differences in age, sex, or MRI scanner manufacturer between the DS group and the neurotypical control group (Table 1).

### 3.2 Effects of age on de‐facing performance

**FIGURE 2:**
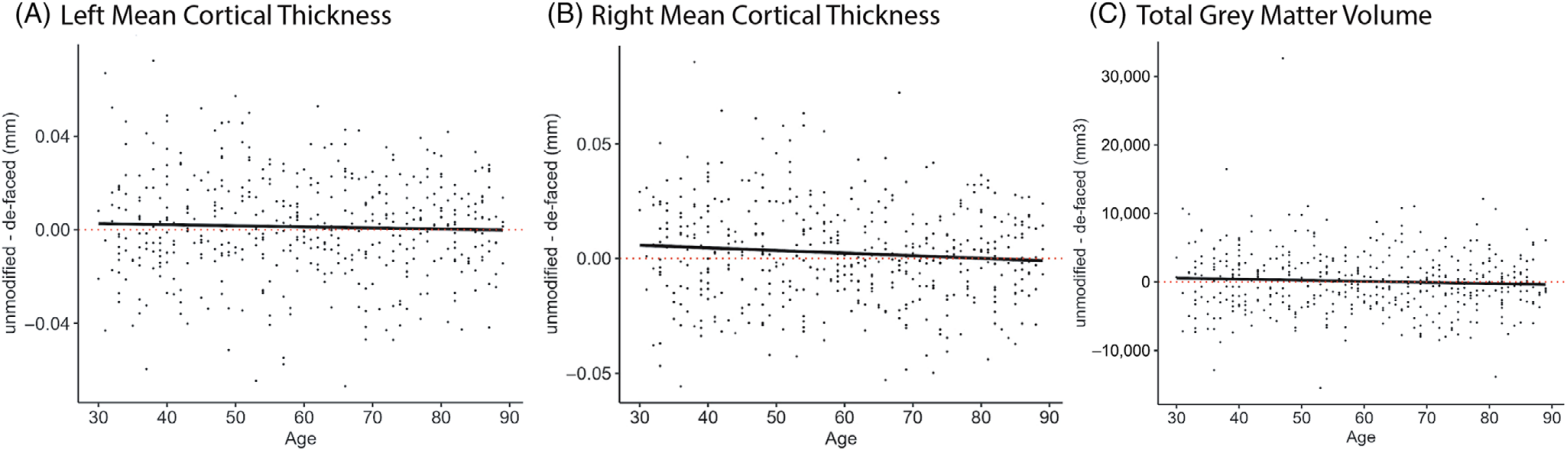
Effect of age on de‐facing with *mri_reface* in neurotypically developed individuals. Shown is the linear relationship between the difference in cortical thickness or gray matter volume before and after de‐facing as a function of age, where a regression line with a gradient of 0 would indicate no age effect on de‐facing. De‐facing with *mri_reface* displayed no age‐related effect on cortical thickness in the left hemisphere (*p *= 0.059) (A), cortical thickness in the right hemisphere (*p* = 0.431) (B), or total gray matter volume (*p* = 0.186) (C). These comparisons also controlled for effects of sex and MRI scanner manufacturer.

The influence of age on de‐facing performance in TD individuals was examined by measuring change in FreeSurfer regional cortical thickness and gray matter (GM) volumes before and after de‐facing. De‐facing with *mri_reface* had no effect on mean cortical thickness in either the left (*p* = 0.059) or right (*p* = 0.431) hemisphere (Figure 2A,B). No change in total GM volume (*p* = 0.186) was observed (Figure 2C). Assessment of 151 FreeSurfer‐defined regional cortical thickness and GM volume measurements revealed 17 regions with nominally significant age‐associated changes in volume following de‐facing. However, none of these effects survived corrections for multiple comparisons (see Table S1 for all regions).

### 3.3 Effects of DS on de‐facing performance

**FIGURE 3:**
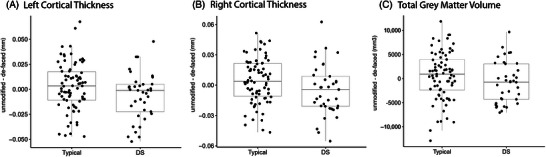
Effect of Down syndrome (DS) on de‐facing with *mri_reface* compared to matched neurotypically developed controls. De‐facing in adults with DS has no effect following correction for multiple comparisons on left cortical thickness (*p *= 0.037, corrected *p* = 0.997) (A), right cortical thickness (*p =* 0.046, corrected *p* = 1.0) (B), or total gray matter volume (*p* = 0.147) (C) compared to the effects of de‐facing with *mri_reface* in neurotypically developed controls (typical). These comparisons also controlled for effects of age, sex, and MRI scanner manufacturer.

The performance of *mri_reface* was assessed in adults with DS compared to a TD control group. When comparing the subtractive differences (original – de‐faced) for each regional FreeSurfer measurement, between the group of adults with DS and the TD adults, a significant difference in mean cortical thickness was observed in the left (*p* = 0.037, corrected = 0.997) hemisphere and right (*p* = 0.046, corrected = 1.0) hemisphere. However, a small effect size, 0.0095 and 0.0092 mm for the left and right hemispheres, respectively, was observed and neither survived corrections multiple comparisons (Figure 3A,B). No difference in the change in total GM volume following de‐facing was observed between the groups (*p* = 0.147) (Figure 3C). Of the 151 FreeSurfer‐defined cortical thickness and GM volume regions assessed, 21 were found to be nominally significantly different. Of these regions, none survived corrections for multiple corrections (see Table S2 for all regions). No difference in failure rate was observed between individuals with DS (2/39) or the MCSA matched group (0/78) (Fisher's exact test, *p *= 0.109).

### 3.4 Effects of de‐facing on DS biomarker performance

**FIGURE 4:**
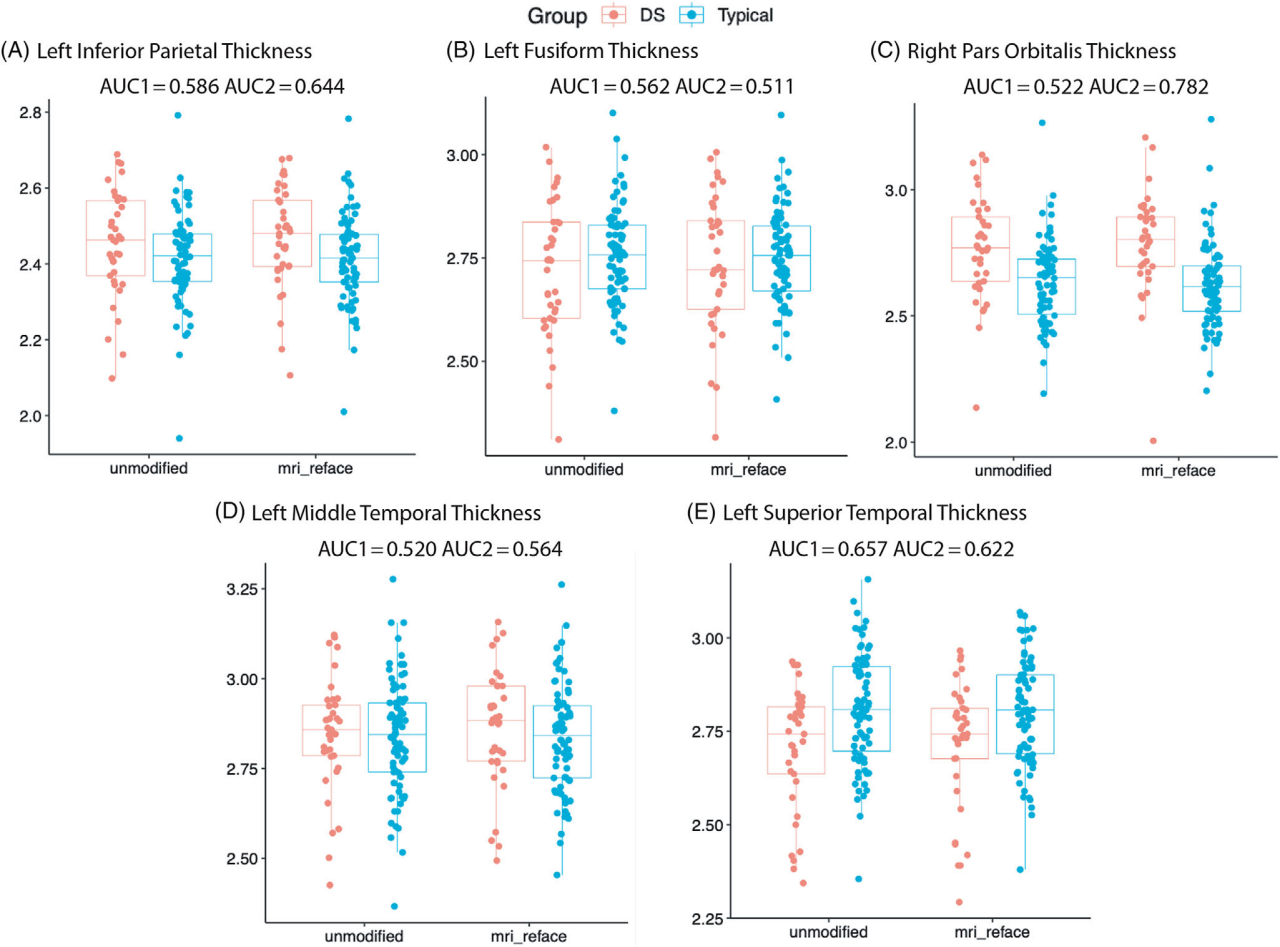
Regions with the largest change in discrimination between Down syndrome (DS) and neurotypically developed individuals following de‐facing with *mri_reface*. Shown are the area under the receiver operating characteristic (AUROC) curve values for the regions that display nominally significant change in AUROC values for discrimination of adults with DS and neurotypically developed individuals (typical) following de‐facing with *mri_reface*; however, none survive correction for multiple comparisons (AUC1; AUROC for unmodified scans, AUC2; AUROC for MRI scans following de‐facing with *mri_reface*). Left inferior parietal thickness (*p* = 0.0022, corrected *p* = 0.0763) (A), left fusiform thickness (*p* = 0.0027, corrected *p* = 0.0973) (B), right pars orbitalis thickness (*p* = 0.012, corrected *p* = 0.514) (C), left middle temporal thickness (*p* = 0.026, corrected *p* = 0.861) (D), and left superior temporal thickness (*p* = 0.029, corrected *p* = 0.889) (E).

**FIGURE 5:**
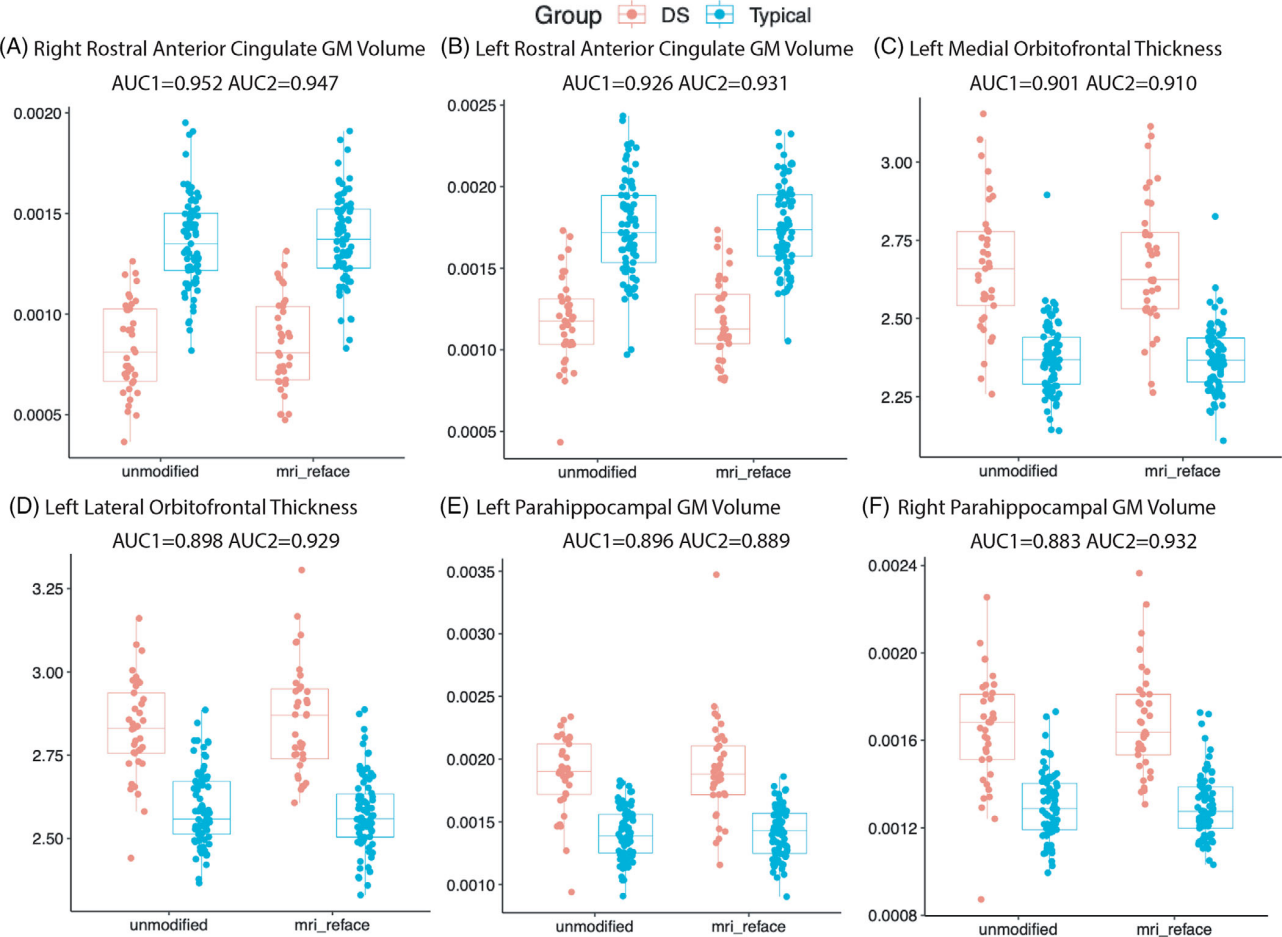
Regions displaying the best discrimination between adults with Down syndrome (DS) and neurotypically developed individuals before and after de‐facing with *mri_reface*. Shown are the area under the receiver operating characteristic (AUROC) curve values before and after de‐facing for the regions that display the largest AUROC values for discriminating adults with DS and neurotypically developed individuals (typical) (AUC1; AUROC for unmodified scans, AUC2; AUROC for MRI scans following de‐facing with *mri_reface*). None of these regions display significant changes following de‐facing with *mri_reface*. Right rostral anterior cingulate gray matter (GM) volume (AUROC value before de‐facing = 0.952, after de‐facing = 0.947) (A), left rostral anterior cingulate GM volume (before de‐facing = 0.926, after de‐facing 0.931) (B), left medial orbitofrontal thickness (before de‐facing = 0.901, after de‐facing = 0.910) (C), left lateral orbitofrontal thickness (before de‐facing = 0.898, after de‐facing = 0.929) (D), left parahippocampal GM volume (before de‐facing = 0.896, after de‐facing = 0.889) (E), right parahippocampal GM volume (before de‐facing = 0.883, after de‐facing 0.932) (F).

To assess the effect of de‐facing with *mri_reface* on potential study outcomes, we assessed whether de‐facing caused measurable changes in AUROC values for classifying DS and TD individuals using each regional measurement. Five of the 151 regions assessed displayed nominally significant differences in power to classify DS versus TD individuals as measured by AUROC: left inferior parietal thickness (*p* = 0.0022, corrected *p* = 0.0763), left fusiform thickness (*p* = 0.0027, corrected *p* = 0.0973), right pars orbitalis thickness (*p* = 0.012, corrected *p* = 0.514), left middle temporal thickness (*p* = 0.026, corrected *p* = 0.861), and left superior temporal thickness (*p* = 0.029, corrected *p* = 0.889) (Figure 4). None of these observed effects survived corrections for multiple comparisons, and all were in regions that were relatively poorly discriminatory with AUROCs < 0.73. There were 44 regions with higher (>0.73) AUROCs that displayed no significant change in AUROC following de‐facing. Regions with the highest AUROC were right rostral anterior cingulate GM volume (before de‐facing = 0.952, after de‐facing = 0.947), left rostral anterior cingulate GM volume (before de‐facing = 0.926, after de‐facing 0.931), left medial orbitofrontal thickness (before de‐facing = 0.901, after de‐facing = 0.910), left lateral orbitofrontal thickness (before de‐facing = 0.898, after de‐facing = 0.929), left parahippocampal GM volume (before de‐facing = 0.896, after de‐facing = 0.889), and right parahippocampal GM volume (before de‐facing = 0.883, after de‐facing 0.932) (Figure 5) (for all regions see Table S3).

## 4 DISCUSSION

In this study, we investigated the validity of a recently developed MRI de‐identification procedure in adults with DS, the first report of de‐facing in individuals with DS. As data sharing of neuroimages becomes increasingly widespread in DS research, it is of vital importance to ensure that data are appropriately de‐identified while maintaining optimal data integrity. This is true in any population, but even more so in vulnerable populations like those with DS. This study reveals that *mri_reface* produces minimal impact on FreeSurfer‐derived MRI volumes and cortical thickness.

### 4.1 Effect of age on de‐facing performance

As AD‐related pathology develops markedly earlier in DS than in sporadic AD, with overproduction of amyloid beta detectable before birth and amyloid plaques detectable on PET by 40 years of age,^9^, ^25^ our study investigated the effects of age and DS on the performance of MRI de‐facing with *mri_reface*. Previous work indicated that in adults 19 to 31 years old and children, some de‐facing methods display an increased likelihood of altering brain voxels. However, *mri_reface* performance on those between 31 and 56 years old was not assessed.^26^ While we observed nominally significant linear effects when assessing the relationship between age and de‐facing‐caused change in regional cortical thickness or volume in TD individuals, no effects survived corrections for multiple comparisons, indicating that although *mri_reface* was developed with an averaged face template from aged populations, it performs well in adults over 30. Further studies are needed to understand the performance of *mri_reface* in populations less than 30.

### 4.2 Effects of DS on de‐facing performance

We compared de‐facing‐related changes in MRI measures between adults with DS and TD individuals. No changes following de‐facing with *mri_reface* survived correction for multiple comparisons. Thus, even though *mri_reface* was originally validated using older individuals without DS, and it replaces faces with an average of individuals without DS, the effects of *mri_reface* on individuals with DS were no larger than for TD individuals. The largest differences were observed in left fusiform thickness, left superior temporal thickness, left lateral orbitofrontal thickness, and right middle temporal thickness. This differs from the original validation studies with *mri_reface* where insula cortical thickness bilaterally and frontal and temporal pole bilaterally (thickness and GM volumes) were found to be the most affected.^16^ These differences may be due to altered craniofacial morphology in adults with DS or the smaller group sizes in the present study. Although the versions of FreeSurfer used between the studies differed (v6.3.0 vs 7.4.1), these version differences are unlikely to underly the observed differences between studies. When assessing differences between de‐facing effects in TD versus DS, global mean thickness in the left and right hemispheres displayed nominally significant effects, which did not survive corrections for multiple comparisons. The average magnitudes of these differences (0.0095 mm [left] and 0.0092 mm [right]) were extremely small, suggesting these changes would have little effect on study outcomes. The scan‐rescan variance for these measures was previously estimated (with FreeSurfer 7.3.2 in older adults) as a median absolute deviation of 0.018 mm (left) and 0.022 mm (right),^4^ so our estimates of average de‐facing‐caused biases in DS are far smaller than estimated scan‐rescan effects, further supporting their negligibility. Individuals with DS displayed a numerically greater failure rate than TD individuals, but this difference was not significant. Across all scans, including those excluded for age and scanner type, the total failure rate was 2/114 (1.75%). Adjusting the TIVtoleranceOffset on *mri_reface* salvaged these scans. It is important to note that altering TIVtoleranceOffset is only recommended for scans where brain voxels are changed as editing TIVtoleranceOffset increases the risk of the eyebrow ridge being retained and the image not being appropriately deidentified.

### 4.3 Effects of de‐facing on DS biomarker performance

The effect of *mri_reface* on how a given region discriminated between individuals with DS and TD individuals was investigated by comparing the AUROC before and after de‐facing for FreeSurfer‐derived measurements. In regions that best distinguished individuals with DS and TD individuals (AUROC > 0.73, *n* = 44 regions), no significant differences were observed before and after de‐facing. In the six regions with the highest AUROCs, the rostral anterior cingulate had a smaller GM volume in participants with DS than TD controls bilaterally, while the left lateral and medial orbitofrontal cortical thicknesses and parahippocampal GM volumes were greater in participants with DS. These results are consistent with previous studies, where non‐demented adults with DS displayed greater cortical thicknesses across posterior and anterior cortical regions compared to TD controls,^27^ as well as reduced GM volume in the cingulate gyrus and larger GM volume in the parahippocampal gyrus.^28^ While none of the previous studies provided a causal factor for these differences, hypothesized factors include abnormal neurodevelopmental processes, response to early AD pathology, and reduced GM and white matter contrast.^27^, ^28^ Only five regions across the 151 regions assessed displayed a nominally significant difference in AUROC; however, none of these effects survived correction for multiple comparisons. Across the whole dataset, the average absolute difference in AUROC was 0.022, indicating a very small change in how well different regions discriminated between individuals with DS and TD individuals following de‐facing with *mri_reface*. These data suggest that the minimal effects of *mri_reface* on different brain measurements are unlikely to influence study outcomes negatively, neither qualitatively nor in terms of statistical power.

### 4.4 Strengths

The greatest strength of our study is that it is the first to investigate whether de‐facing software developed for older TD individuals can be used on images from people with DS, without introducing non‐biological differences. Data from individuals with DS are extremely valuable to AD research because they have a genetically determined form of AD with almost complete penetrance, representing an opportunity to learn how AD pathology develops through the lifespan. However, individuals with DS are a vulnerable and relatively small population, warranting stringent privacy protections, challenging broad sharing of research data for the greater scientific good. Our study shows that a leading, widely used de‐facing software can be applied to neuroimaging data from adults with DS, removing a major barrier to sharing of these research data.

### 4.5 Limitations

One limitation of the methodology used is that individuals in the DS versus TD comparisons were balanced on scanner manufacturer, not manufacturer and model, due to non‐overlap between the scanner models across MCSA and TRC‐DS. In the final analyses, the groups were no longer completely matched 1:2 DS to neurotypical controls; however, they were still very well balanced on the group level (Table 1). It is important to note that other tools, such as FMRIB Software Library (FSL) and Statistical Parametric Mapping (SPM12), are available to derive brain volumes and cortical thickness, and they may perform differently following de‐facing. Furthermore, de‐facing is an important consideration across most brain MRI sequences (diffusion MRI and functional MRI currently being notable exceptions), CT, and PET imaging,^1^, ^2^, ^3^, ^4^ and performance may differ by modality or sequence. However, in TD individuals, *mri_reface* has been demonstrated to perform similarly across numerous different neuroimaging analysis platforms^8^, ^16^ and imaging modalities^1^, ^4^, ^8^; as such, the minimal effect on structural neuroimaging parameters observed following de‐facing of T1‐weighted MRI scans in adults with DS suggests *mri_reface* will perform well across neuroimaging modalities in adults with DS. We also only tested *mri_reface*, despite the existence of many de‐facing software programs, because *mri_reface* is used in a number of large AD imaging cohorts,^8^ and some previous comparisons between de‐facing software applications have demonstrated superior performance.^7^, ^8^, ^16^ For the assessment of biomarker performance, volumes were adjusted by dividing by intracranial volume, which can introduce bias.^29^, ^30^ This was done as covariate‐based adjustments are not readily incorporated into AUROC‐based assessments. This study investigated the utility of *mri_reface* in adults with DS from the age of 30, but further studies will be needed to assess the performance of *mri_reface* in younger individuals with and without DS. We did not directly test the performance of commercial facial recognition systems on DS individuals after de‐facing due to regulatory (consent) constraints; however, visual assessment confirmed that facial features including the eyebrow ridge were successfully replaced, which, based on previous studies, would provide sufficient deidentification.^16^ Philips scanner performance was not directly assessed; however, the failure rate of DS participants scanned on Philips was low (0/38), and previous validation of *mri_reface* included participants acquired on Philips scanners.^1^, ^8^

## 5 CONCLUSION

In conclusion, *mri_reface* provides a valid method for the de‐identification of neuroimages in adults with DS and should be considered for use in future and ongoing studies in adults with DS where data are shared with the broader scientific community. These findings support the robustness of the *mri_reface* pipeline and that downstream brain volumetrics are not significantly affected by replacing DS faces with TD faces. This will facilitate more widespread sharing of de‐identified imaging data in adults with DS, enabling more AD research studies in a vulnerable population who develop AD pathology from birth and display early‐onset dementia, resulting in fewer age‐related co‐pathologies compared with sporadic AD.

## CONFLICT OF INTEREST STATEMENT

Jason K. Russell and Michael S. Rafii are employed by the University of Southern California and the Alzheimer's Therapeutics Research Institute (ATRI). Michael S. Rafii has received grants or contracts from Eisai and Eli Lilly, which were paid to his institution. He has received consulting fees from AC Immune and Ionis. He has participated on a data safety monitoring board or an advisory board for Alzheon, Alnylam, Biohaven, Embic, Prescient Imaging, Positrigo, and Recall Therapeutics. Walter K. Kremers, Scott A. Przybelski, Prashanthi Vemuri, Ronald C. Petersen, Jonathan Graff‐Radford, Clifford R. Jack, and Christopher G. Schwarz are employed by the Mayo Clinic. Walter K. Kremers, Prashanthi Vemuri, Ronald C. Petersen, Jonathan Graff‐Radford, Clifford R. Jack, and Christopher G. Schwarz receive grant funding from the National Institutes of Health, related and unrelated to this study, paid to their institution. Ronald C. Petersen received consulting fees from Roche, Genentech, Eli Lilly, Eisai, Novo Nordisk, and Novartis and receives royalties from Oxford University Press and UpToDate. Clifford R. Jack received funding from the GHR Foundation and Alexander Family Alzheimer's Disease Research Professorship of the Mayo Clinic. Author disclosures are available in the Supporting Information.

## CONSENT STATEMENT

All participants or their legally authorized representatives for the study gave written informed consent in accordance with the Declaration of Helsinki and approved Institutional Review Board protocol.

## Supporting information



Supporting Information

Supporting Information

Supporting Information

Supporting Information
