# Supplementary material for: Facial feature removal in magnetic resonance imaging scans of adults with Down syndrome: A de‐facing methodological study
Source: Alzheimers Dement. 2026 Jun 26;22(7):e71614. doi: 10.1002/alz.71614 (PMC13307342; doi:10.1002/alz.71614)
Supplement: Supplementary file 1 — Supporting Information [file ALZ-22-e71614-s003.docx]

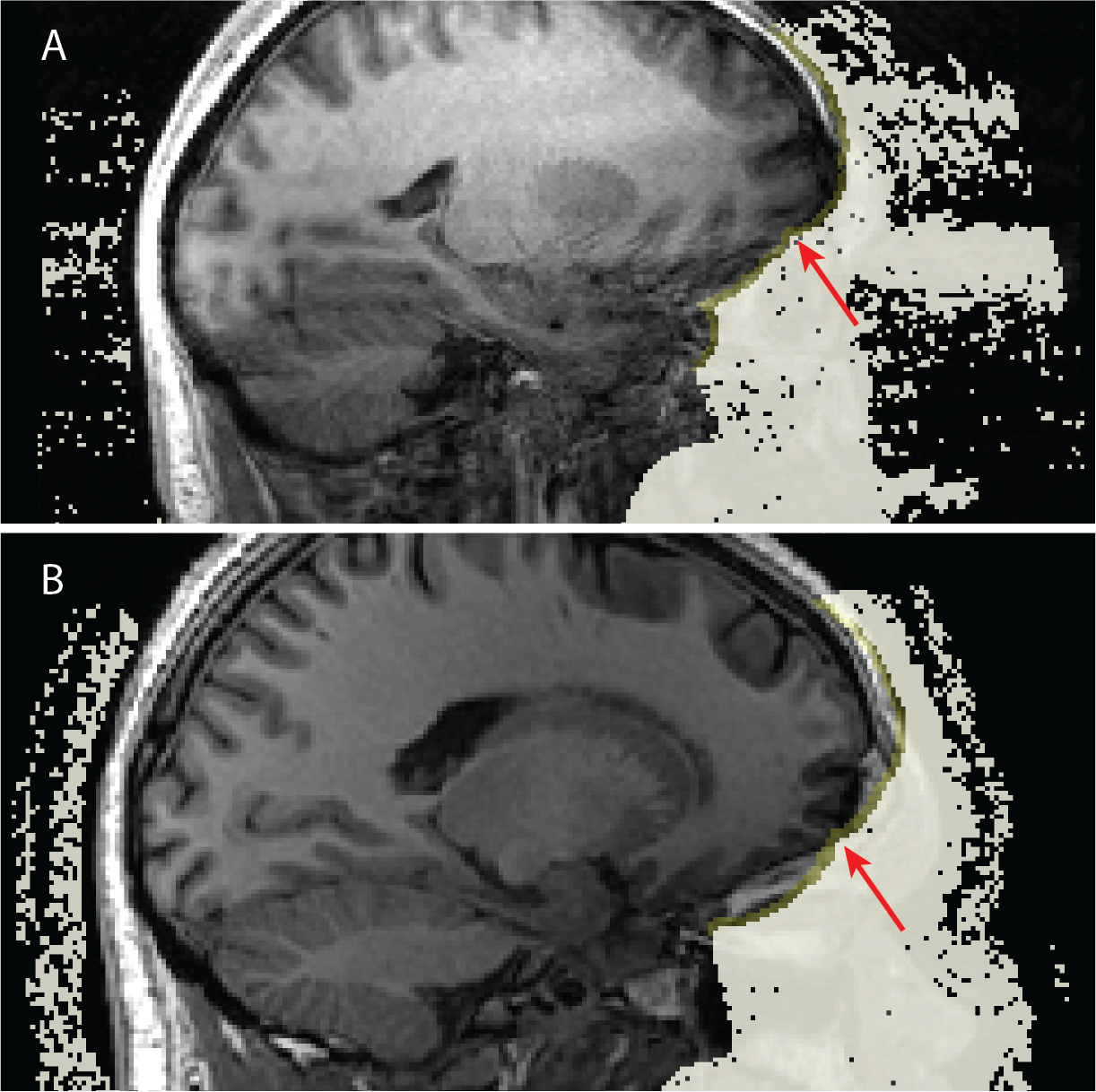


**Supplementary Figure 1.** Shown are the two participants with Down syndrome that initially failed quality control. The initial changed voxels are shown in pale yellow, with the adjusted defacing (with -TIVToleranceOffset +2) shown in white. The red arrow indicates the region where brain tissue was altered prior to correction. The eyebrow ridge and facial features were still appropriately edited by *mri_reface* following the adjustments to TIVTolerance.
